# Sex-dependent prediction of autism

**DOI:** 10.3389/fgene.2026.1799530

**Published:** 2026-08-13

**Authors:** Catriona J. Miller, Theo Portlock, Denis M. Nyaga, Justin M. O’Sullivan

**Affiliations:** 1 The Liggins Institute, The University of Auckland, Auckland, New Zealand; 2 The Maurice Wilkins Centre, The University of Auckland, Auckland, New Zealand; 3 MRC Lifecourse Epidemiology Unit, University of Southampton, Southampton, United Kingdom; 4 Singapore Institute for Clinical Sciences, Agency for Science Technology and Research (A*STAR), Singapore, Singapore

**Keywords:** autism, common variants, machine learning, sex-dependence, whole genome sequencing

## Abstract

**Introduction:**

Autism spectrum disorders (ASD) have a global prevalence of 1%, with a male-to- female diagnosis ratio of roughly 4:1. Several models have been developed to predict ASD using genetic information. However, the influence of biological sex on prediction outcomes remains underexplored.

**Methods:**

We present an ensemble model to predict ASD, which integrates polygenic risk scores (PRSs), common genetic variants, and ASD risk genes with the MSSNG whole genome sequencing (WGS) dataset.

**Results:**

Following training, our model achieved an accuracy of 0.68, an area under the receiver operating curve (AUROC) of 0.72, and a recall of 0.77 on the test dataset. Notably, common variants contributed more significantly to ASD prediction in males than females (p < 0.001), with accuracies of 0.69 and 0.66, respectively. The 16p11 locus emerged as particularly predictive for females (p < 0.001). Gene enrichment analysis using the Allen Brain Atlas revealed that expression of ASD risk genes that were significant in females were enriched (FWER < 0.05) in the primary somatosensory cortex, inferior parietal cortex, and parietal neocortex during fetal development. By contrast, male ASD risk gene expression was enriched (FWER < 0.05) in the dorsolateral prefrontal cortex and anterior cingulate cortex across developmental stages (fetal to adult).

**Discussion:**

These findings underscore a sex-dependent role for common genetic variants in the risk of developing ASD. In doing so, they highlight the utility of ensemble models that incorporate common variation and biological sex for ASD prediction.

## Introduction

Autism spectrum disorders (ASD) are heterogeneous and characterised by challenges with social skills, repetitive behaviours, and communication deficits. ASD is highly heritable, with a twin-based genetic heritability of 64%–91% (Tick et al., 2016). Like other neuropsychiatric and neurodevelopmental traits, common variants have a low individual impact but collectively contribute additively to the heritability of ASD (Grove et al., 2019). In contrast, rare variants have a high individual impact but a low overall effect on liability (Gaugler et al., 2014).

ASD has a global prevalence of 1%. However, the prevalence varies across regions and demographics (Zeidan et al., 2022). Notably, there are roughly four times as many diagnosed males as females (Bougeard et al., 2021; Zeidan et al., 2022). Different hypotheses have been proposed to explain the ‘female protective effect’. These hypotheses include the liability threshold model, which suggests that females have a higher threshold for diagnosis (Dougherty et al., 2022). The liability threshold is thought to be influenced by both genetic (e.g., sex chromosome and methylation differences) and non-genetic (e.g., diagnostic bias and hormones) factors (Dougherty et al., 2022). Failure to account for the female protective effect during model development and deployment may introduce significant bias into research outcomes (Cirillo et al., 2020). However, including biological sex as a variable in attempts to predict ASD offers the potential to elucidate the genetic contributions of the putative protective effect.

The use of genetic models to predict ASD and other neuropsychiatric conditions has had mixed success (Bracher-Smith et al., 2021; Gupta et al., 2022). Early methods used polygenic risk scores (PRS) as a logistic regression for prediction (Benca et al., 2017; Jansen et al., 2020). However, the last decade has witnessed a substantial increase in the application of artificial intelligence (AI) and machine learning techniques (Manduchi et al., 2022; Novakovsky et al., 2023). Incorporating whole genome sequencing into prediction models has also improved results, in part through the inclusion of non-coding regions, which are recognised as having a role in determining traits (Elkon and Agami, 2017; Rosenquist et al., 2022). Finally, machine learning approaches have been applied to the genetics of neurodevelopmental conditions for diagnosis, condition subtyping, variant identification, and biomarker prioritisation (Gupta et al., 2022).

The accuracy of predicting the probability of being Autistic from genetic sequencing datasets has ranged from 0.52 to 0.81 (Bracher-Smith et al., 2021). However, the majority of the models that have been used for predictions are limited due to either: 1) a lack of separate validation data (Ghafouri-Fard et al., 2019), 2) using a small, homogeneous population (Wang et al., 2018), or 3) lack of breakdown of predictability between sex and limited reporting of results (e.g., accuracy only) (Bracher-Smith et al., 2021).

In this study, we developed a robust ensemble model to predict the probability of being Autistic using common genetic variation, leveraging the Autism Speaks’ MSSNG whole-genome sequencing dataset comprising 11,000 individuals (Trost et al., 2022). Our approach identified key genetic variants and genes contributing to ASD probability and their sex-dependent effects.

## Materials and methods

### Data preparation

Controlled access to the MSSNG database was applied for and approved by the MSSNG Database’s Data Access Committee (DACO-2020–04). MSSNG consists of whole-genome sequences from 5,102 Autistic individuals (4,074 males, 1,028 females), 6,079 unaffected individuals (3,033 males, 3,046 females), and 131 ASD-related individuals (those with an ASD-related diagnosis; 61 males, 70 females) (Trost et al., 2022). Of these, 1,732 individuals’ samples were sequenced on Complete Genomics (CG), and 9,580 were sequenced on Illumina HiSeqX platforms. Database demographics in Supplementary Table S1 and in Trost et al. (2022). The 131 ASD-related individuals were removed from the dataset before quality control.

Quality control was performed separately on the CG and Illumina datasets using PLINK (Version 1.9) (Purcell et al., 2007). SNPs with a missing rate above 5% or a Hardy-Weinberg equilibrium (HWE) p-value below 10^–6^ were removed. Additionally, we filtered out: (1) individuals with a missing rate above 5%, (2) outlying homozygosity (more than three sd from the mean level of homozygosity), (3) ancestry outliers (more than three sd away from principal components 1 and 2’s cluster mean), and (4) similar samples (an identity by descent [IBD] above 0.5). Y-chromosome variants were also removed. After quality control, 1,533 individuals and 10,615,362 variants remained from the CG dataset, and 8,124 individuals and 10,116,316 variants remained from the Illumina dataset. Full quality control methods available at https://github.com/Catriona-Miller/autism_ml.

### Feature selection

**FIGURE 1:**
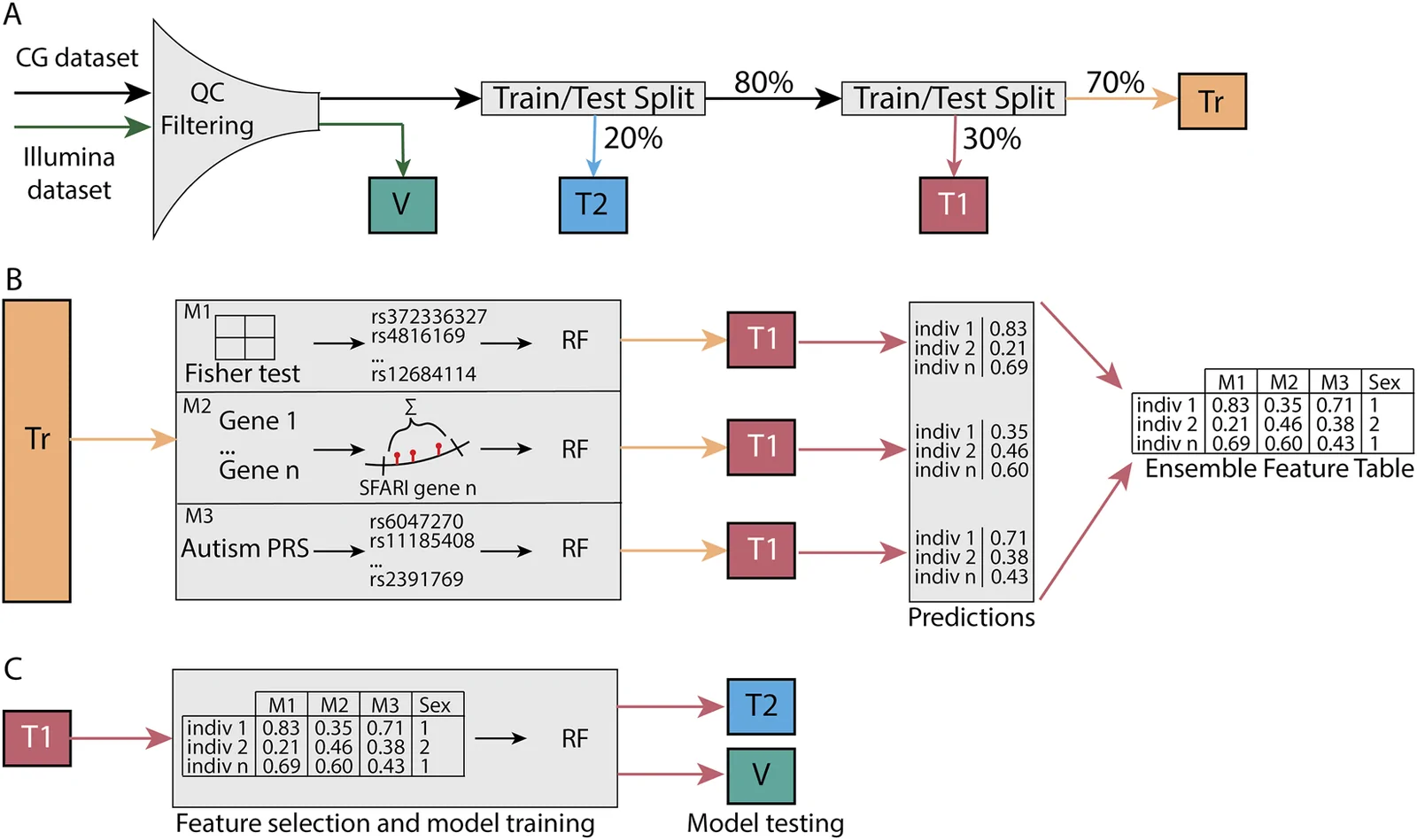
Schematic of the methods used to train the ensemble ML model. **(A)** Datasets were split into train/test following quality control (QC). Illumina data was retained for validation (V). Complete genomics (CG) data was split into training (Tr) and two test sets (T1 and T2). **(B)** Random forest models were trained on 1) variants selected by a Fisher test on the training data, 2) normalised mutation rates per SFARI gene, and 3) variants from an autism PRS (Grove et al., 2019). **(C)** The predictive scores from the models trained in B and biological sex were used to train the final ensemble random forest model. RF: random forest.

The CG dataset was used for model training and testing. A 56:24:20 split (training: test 1: test 2) was applied to the CG dataset. The 56% training dataset was used to train 1) a model using a large number of SNPs (790) identified as statistically significantly associated with ASD in the training dataset, 2) a model using ASD-associated genes, and 3) a model using a small number of SNPs previously shown to be associated with ASD (40) (Figure 1). The larger Illumina dataset was reserved as an external validation set. While training on the smaller subset is a more conservative approach that potentially limits maximum model performance, it increases confidence that any signal detected is robust enough to generalise to a much larger cohort.

### Model 1 – Fisher SNPs

The input for this process was the 10,615,362 variants remaining from the CG dataset following quality control. To perform feature selection, the 56% training dataset first underwent LD pruning (50 variant count [vc] window size, 5 vc step size, 2 variance inflation factor [VIF] threshold; using PLINK’s *indep* function (Purcell et al., 2007)). This sliding variant count window was employed primarily as a dimensionality reduction technique to remove highly correlated features and improve the model’s performance.

Following LD pruning, a Fisher exact test was performed on the remaining, relatively independent set of SNPs to identify those associated with ASD. We selected a p-value threshold of < 5 × 10^−5^ (Supplementary Table S2). As the primary objective of this step was feature selection, the conventional GWAS threshold of 5 × 10^−8^ was deemed overly stringent. A threshold of 5 × 10^−5^ was chosen to balance retaining enough informative features (790 SNPs; FisherSNPs on https://github.com/Catriona-Miller/autism_ml) without including too many. The feature selection pipeline is outlined in Supplementary Figure S1. A feature table with these SNPs (0,1,2; AA, Aa, aa, respectively) was fed into a random forest model (PyCaret version 3.3.2). Ten-fold cross-validation was performed for hyperparameter optimisation in PyCaret (version 3.3.2) (Ali, 2020) using the 56% training dataset. The model was then tested on the 24% test 1 dataset.

### Model 2 – SFARI genes

A list of 1,037 genes associated with ASD was downloaded (2024/03/28) from the SFARI database (Abrahams et al., 2013) (Supplementary Table S3). The number of variants per gene was calculated for each individual and formed the feature table. Ten-fold cross-validation was performed for hyperparameter optimisation in PyCaret (version 3.3.2) on the 56% training dataset. The model was then tested on the 24% test 1 dataset.

### Model 3 – GWAS SNPs

A list of 40 SNPs associated with ASD (Grove et al., 2019) was used to create a feature table (0,1,2; AA, Aa, aa, respectively) and fed into a random forest model (Supplementary Table S4). Ten-fold cross-validation was performed for hyperparameter optimisation in PyCaret (version 3.3.2) on the 56% training dataset. The model was then tested on the 24% test 1 dataset.

### Ensemble model development

The prediction scores from the three models alongside biological sex were used to form a feature table (https://github.com/Catriona-Miller/autism_ml). A random forest model was trained on the 24% test 1 data (ten-fold cross-validation) and then tested on the 20% test 2 data. This model was then validated using the Illumina data.

### Feature contribution analysis

Shapley additive explanations (SHAP) values were used to analyse how each model and the features within these models contributed to the overall prediction (Lundberg et al., 2020). SHAP is a game-theoretic approach that calculates the contribution of each feature to the overall model prediction (Shapley, 1953; Lundberg and Lee, 2017). The Python SHAP package (version 0.46) was used to calculate SHAP values for each model and the overall ensemble model (Lundberg et al., 2020).

### Developmental enrichment analysis of SFARI genes

A Mann-Whitney U test was performed to compare the number of mutations in the set of genes previously associated with ASD (all SFARI genes) between Autistic and neurotypical individuals for females and males separately. Multiple tests were corrected for using the false discovery rate (Benjamini Hochberg test). All genes that had a statistically significant difference (p-value < 0.05) in variants across their gene length between Autistic and neurotypical individuals were referred to as male and/or female SFARI genes.

RNA-Seq data from 42 individuals’ brains at five developmental stages (prenatal, infant, child, adolescent, adult) were obtained from the Allen Brain Atlas (ABA) and analysed to identify the brain regions and developmental time points in which the male and female SFARI gene sets’ expression was enriched (Sunkin et al., 2012). Briefly, ABAEnrichment (version 1.2.2) was employed to perform a developmental enrichment analysis (Grote et al., 2016). Genes were annotated to a brain region if the expression was above the 0.8 cutoff (Grote et al., 2016). A hypergeometric test was used to identify brain regions and developmental time points where the annotated gene list was enriched in the male or female SFARI gene list, compared to background control genes. A family-wise error rate (FWER) was calculated by comparing the enrichment against 1,000 random sets of equal size (Grote et al., 2016). Brain regions with an FWER < 0.05 were deemed to be significantly enriched. Brain regions that were enriched across development were identified using ABA’s developmental effect score dataset, which contains gene age effect scores based on developmental gene expression changes (Grote et al., 2016).

### Clustering analysis of GWAS SNPs

SHAP values were generated to use as features for clustering by rerunning the GWAS random forest model on the full CG dataset. A test dataset was not required as the goal was to analyse the clusters produced, not to use them for prediction. A Uniform Manifold Approximation and Projection (UMAP) was performed to reduce the SHAP value data to two dimensions. After visualisation, k-means clustering was used to produce six clusters. To analyse the ASD PRS for each cluster, GWAS SNP odd ratios (ORs) were downloaded (https://www.ebi.ac.uk/gwas/efotraits/EFO_0003756 accessed 2024/09/26). PRS was calculated using PLINK’s ‘score’ function (Purcell et al., 2007). A pairwise Tukey test was used to compare the mean PRS z-scores between clusters (Tukey, 1949).

### Data availability

Supplementary Table S5 lists the datasets and software that were used in our analyses.

All scripts are available on GitHub (https://github.com/Catriona-Miller/autism_ml).

## Results

### An ensemble model that incorporates biological sex has the greatest accuracy when predicting ASD

**FIGURE 2:**
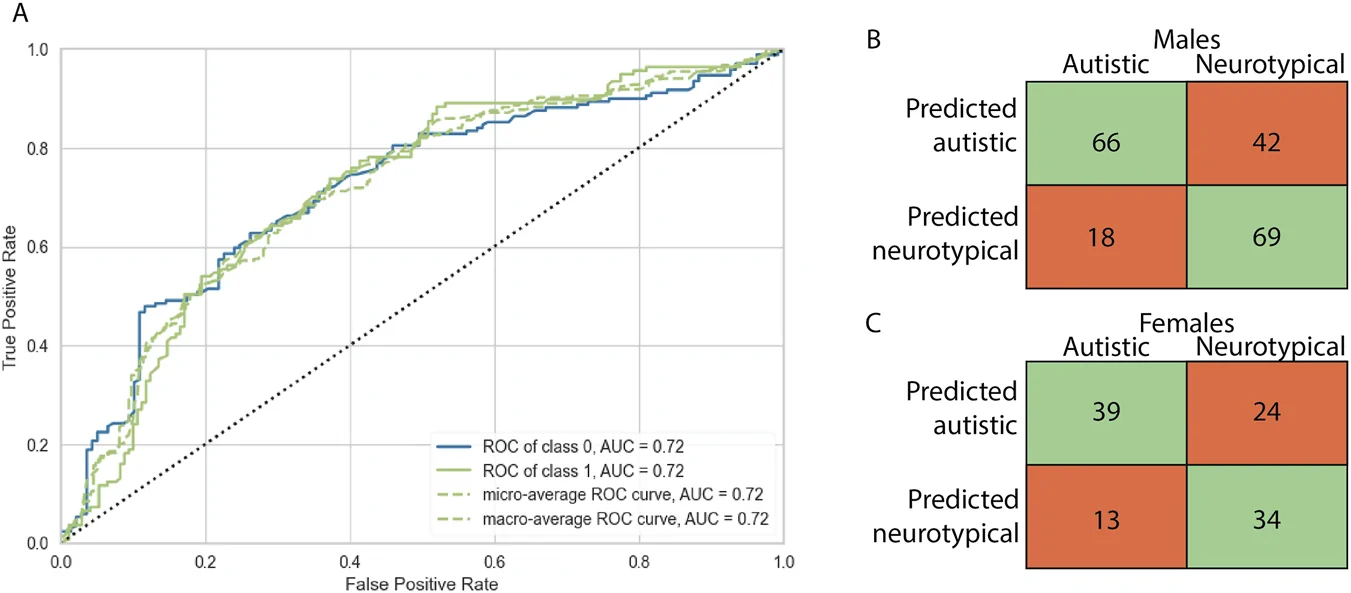
An ensemble model incorporating biological sex predicts autism probability in a previously unseen dataset. **(A)** AUROC graph showing an AUC of 0.72 on the CG test dataset. **(B,C)** Confusion matrix showing the predictions in the Complete Genomics test dataset, split into males and females respectively. Red squares highlight the incorrect predictions (false positives and false negatives) while green highlights the correct predictions (true positives and true negatives).

Random forest models were trained on three independent feature sets (SFARI genes, GWAS, and Fisher SNPs), with the Fisher SNPs model reaching the highest recall (0.86) and the GWAS model reaching the highest accuracy (0.64) when applied to the CG test dataset (Table 1). By creating an ensemble model that combined these three models alongside sex, an accuracy of 0.68 and an AUC of 0.72 were achieved on the test dataset (Table 1; Figure 2A). Recall (the percentage of Autistic individuals correctly predicted to be Autistic) was higher than precision (the percentage of individuals predicted to be Autistic who are Autistic) at 0.77 and 0.61, respectively (Supplementary Figure S2). When analysing predictions by sex, the model achieved accuracies of 0.69 (M) and 0.66 (F) (Figures 2B,C). When applied to the Illumina validation dataset, overall accuracy and AUC saw a moderate reduction compared to the internal test set (Accuracy: 0.63 vs. 0.68; AUC: 0.66 vs. 0.72), while recall remained high (0.79). Because the validation can only use features shared by both platforms, we re-evaluated the finalised ensemble on the CG test set using the reduced feature set (86% of Fisher SNPs; PRS and gene models remained the same). On this matched feature set, CG performance (accuracy 0.65, AUC 0.71, recall 0.77) was close to that on Illumina (Table 1), indicating that the reduction relative to the full-feature model is driven largely by the smaller shared feature set rather than by platform differences.

**TABLE 1:** The ensemble model incorporating biological sex outperforms sub-models for predicting autism probability.

Test dataset	Accuracy	AUC^a^	Recall^b^	Precision^c^	F1^d^
SFARI genes	0.56	0.56	0.37	0.51	0.42
PRS	0.64	0.66	0.8	0.57	0.67
Fisher SNPs	0.63	0.66	**0.86**	0.56	0.68
Ensemble	**0.68**	**0.72**	0.77	**0.61**	**0.69**
Ensemble (CG test, shared SNPs)	0.65	0.71	0.77	0.58	0.66
Ensemble (Illumina validation)	0.63	0.66	0.79	0.56	0.66

Metrics are presented for the models’ predictability on the 20% test set of the Complete Genomics data. The second-to-last row shows the metrics when the CG test set was re-evaluated using only the SNPs present in the Illumina dataset. The final row shows the ensemble model applied to the Illumina validation dataset. a, AUC (area under the curve) represents the probability that a randomly chosen positive (Autistic) instance ranks higher than a randomly chosen negative (neurotypical) instance; b, recall is the percentage of Autistic individuals correctly predicted to be Autistic; c, precision is the percentage of individuals predicted to be Autistic who are Autistic and d, F1 is the harmonic mean of recall (b) and precision (c). For each metric, the score of the model that performed the best is bold.

### Common variants make a large, sex-dependent contribution to the model

The prediction scores from the three models are values between 0–1, quantifying the confidence the model has in the individual being neurotypical (0: ‘certain’ to 0.5 uncertain) or Autistic (0.5 uncertain to 1 ‘certain’). The three prediction scores from the a) SFARI genes, b) GWAS, and c) Fisher SNPs models) were incorporated into the ensemble model alongside sex and, from here on, will be referred to as features.

**FIGURE 3:**
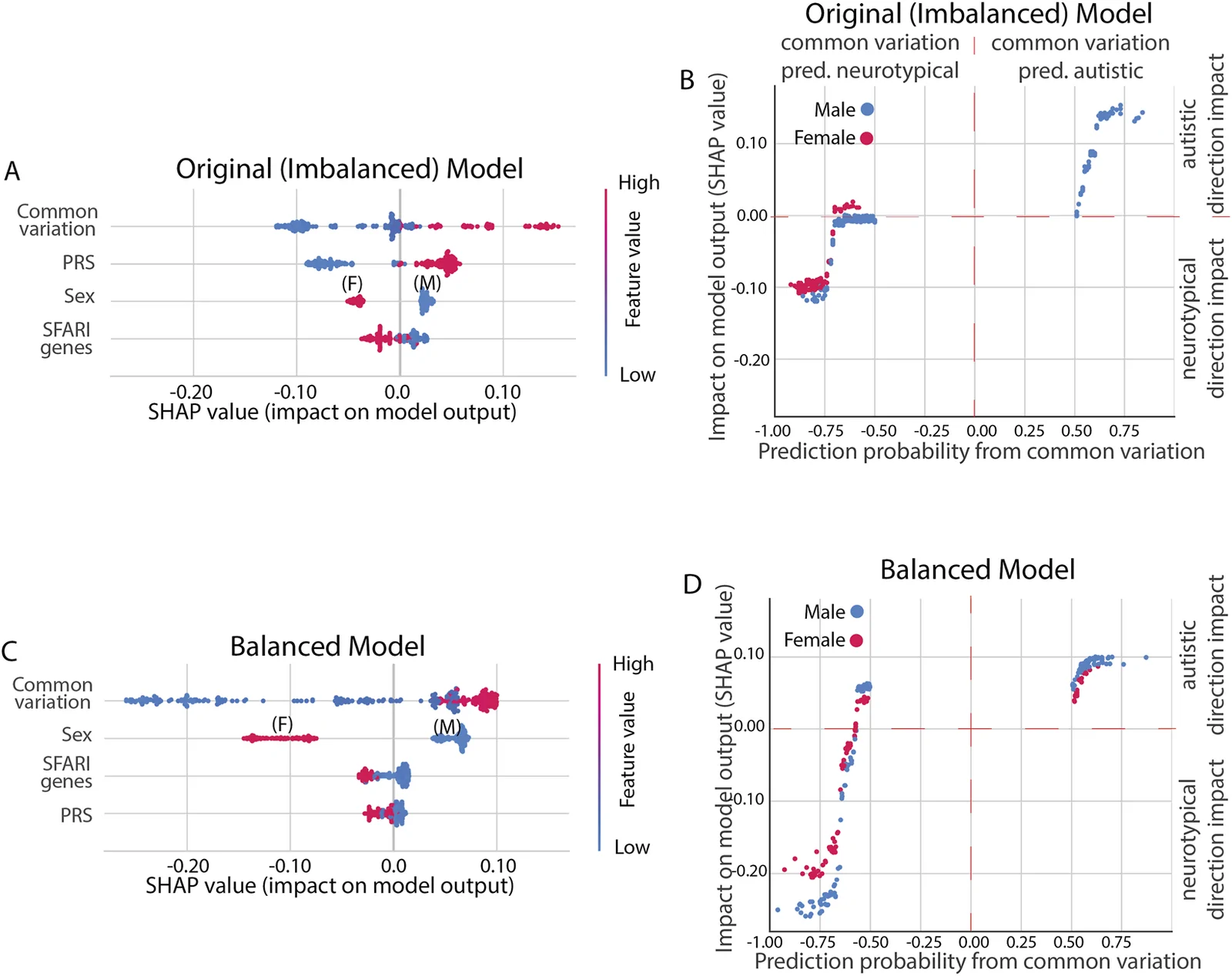
Common variation is an important, sex dependent, predictor in the original and balanced models. **(A,C)** The SHAP values showing the impact of each sub-model (common variation–Fisher’s test, PRS, and SFARI genes) and sex on the overall model. This illustrates the contribution of each sub-model/feature to the overall prediction. A is for the original model while C is the model balanced by sex, i.e., same number of males and females. **(B,D)** Interaction between common variation and biological sex for the original and balanced models, respectively. X-axis shows the prediction scores from the common variation sub-model (fed into the ensemble model as feature values) and the y-axis shows the impact on the overall prediction. Anything left of the dotted line (x = 0.0) would have been predicted as neurotypical by common variation alone while anything above the dotted line (y = 0.0) has a positive impact on the model.

SHAP scores were used to predict the contribution of the four features to the overall ensemble model. The Fisher SNPs model had the highest impact on the ensemble model predictions, followed by the GWAS model, then sex (Figure 3A). This is expected given that the Fisher SNPs model had the highest metric scores of the three individual models (Table 1). We tested for a sex-dependent correlation between Fisher SNP prediction scores and impact on the final prediction (SHAP value) (Figure 3B). We identified a clear relationship between sex and Fisher SNP prediction value in the model, with only males predicted to be Autistic from the Fisher SNP model. For a male and a female with the same neurotypical Fisher SNP predictive value, the ensemble model places a higher weight on common variants for neurotypical males than females (Figure 3B).

The Fisher SNPs model was retrained after balancing the number of males and females within the training dataset. Autistic males within the training dataset were randomly subsampled to match the number of Autistic females. The balanced model had slightly lower scores across all metrics than the original model, with an AUC of 0.70 (Supplementary Table S6). This is likely due to the reduced population size. The Fisher SNPs model prediction values remained the primary driver of classification. However, sex was the second most influential variable in the model’s decision-making process (Figure 3C). This high SHAP value suggests that the model relies heavily on sex to contextualise the genetic variant inputs, effectively treating male and female predictions as distinct risk distributions. In addition, plotting the individual Fisher SNP prediction values against their model impacts (i.e., SHAP values) and colouring by sex, highlights sex-dependent impacts of common variants on prediction (Figure 3D). The balanced model retains greater confidence in male predictions than female predictions from common variants (Mann-Whitney U p-value < 0.001). Female SHAP scores tended toward zero while male SHAP scores tended toward larger values in both directions (negative: neurotypical, positive: Autistic). These results demonstrate the model’s high confidence in predicting ASD probability for males compared to females based on common genetic variants. Thus, despite the balanced sex distribution in our dataset, the model relied more heavily on male-specific genetic risk factors to make its predictions. This suggests that the available common variant signals in this dataset may be more predictive for males than females.

### Top contributing common variants included sex-specific and shared variants

**FIGURE 4:**
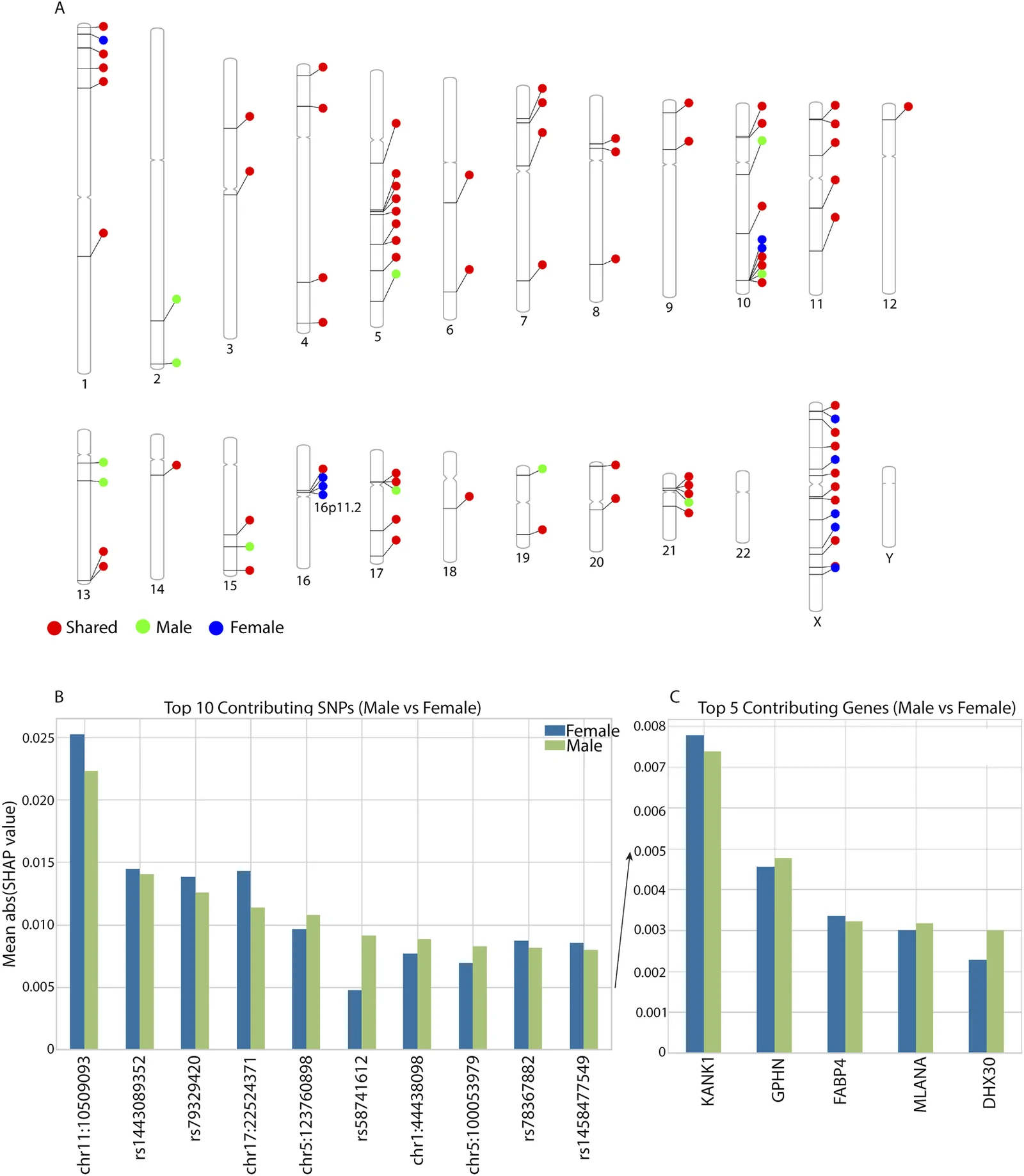
Shared and sex-dependent common variation plays a role in autism prediction. **(A)** Phenogram showing the SNPs with the top 10% of mean absolute SHAP scores for females and males. Those that were in the top 10% of both sexes are red while those only present in the top 10% of females or males are blue and green respectively. **(B)** The mean absolute SHAP values of the top 10 variants, ordered by male SHAP scores, from the Fisher SNPs. **(C)** The mean absolute SHAP values of the top 5 SFARI genes.

Variant contributions to the balanced Fisher model were ranked according to their mean absolute SHAP score for females and males separately, and the top 10% of variants for each sex were selected (Figure 4A). Most variants (67/89, 75%) in the top 10% were shared between males and females.

The top 10 Fisher variants (Figure 4B) and genes (Figure 4C) which contributed to the prediction had similar mean absolute SHAP values in males and females. However, SHAP values for rs58741612 were significantly different between sexes (t-test p-value < 0.001). Rs58741612 (MAF 0.20 in males and females; European: 0.19, African: 0.28, Asian: 0.125) (Karczewski et al., 2020)) has not previously been associated with neurodevelopment. By contrast, the top-ranked variant, chr11:10,509,093, falls within *MTRNR2L8* which has been shown to be fivefold upregulated in Saudi Autistic individuals (Almandil et al., 2022). The second-ranked variant (i.e., rs1443089352) is located within an intron of *P4HA3,* which was shown to be differentially expressed within mildly Autistic individuals (Lee and Hu, 2020).

Notably, there were male- (11/89, 12%) and female-specific (11/89, 12%) loci (Figure 4A). More sex-specific variants were observed in the original (imbalanced) model, likely reflecting a bias towards sex in the original Fisher model (Supplementary Figure S3). 16p11.2 was identified as a female specific locus for ASD risk in both the original and balanced models (t-test p-value < 0.001).

### Male- and female-specific SFARI genes were significantly expressed during different brain developmental windows

We performed sex-stratified analyses comparing mutation rates in SFARI genes between Autistic and neurotypical individuals across males and females separately. This revealed statistically significant differences in mutation rates in 35 genes specific to females, 455 genes specific to males, and 124 genes shared between both sexes (Supplementary Figure S4A; Supplementary Table S7; Supplementary Table S8). For instance, *SHANK3*, one of the most extensively studied SFARI genes (Monteiro and Feng, 2017), was identified as having significantly different mutation rates in the male cohort. The presence of such well-characterised genes in the sex-specific lists highlights the value of stratifying by biological sex. These genes are mapped across different regions of the genome. Unsurprisingly, a large percentage of the female (9/31; 29%) and shared (10/48; 21%) gene sets were located on the X chromosome (Supplementary Figure S4B-D).

Notably, both male- (male + shared; 579) and female-specific (female + shared; 159) sets demonstrated significantly lower (p < 0.001) loss-of-function observed/expected upper bound fraction (LOEUF) scores compared to control SFARI genes without differential mutation rates (Supplementary Figure S4E-F). These findings indicate that these sex-specific genetic contributors are under stronger selective constraint, as reflected by their depletion of loss-of-function variation, underscoring their importance in neurodevelopment.

The Allen Brain Atlas (Sunkin et al., 2012) was used to identify the enrichment of sex-specific gene sets within the brain across developmental timepoints. Expression of genes within the female-specific set was enriched in the primary somatosensory cortex, inferior parietal cortex, and the parietal neocortex during the fetal timepoint (FWER = 0.013, 0.014, 0.010 respectively) (Supplementary Figure S4G). By contrast, expression of genes within the male gene set was enriched (FWER < 0.05) in multiple brain regions at most developmental time points (fetal, infant, child, and adult).

Significant developmental changes in gene expression (i.e., across all developmental windows) were observed for the expression of the male gene set within the dorsolateral prefrontal cortex and the anterior cingulate cortex (FWER = 0.048 and 0.006, respectively). By contrast, there were no significant developmental changes in expression in the female gene set (Supplementary Figure S4G).

### ASD PRS scores can be used to cluster individuals

**FIGURE 5:**
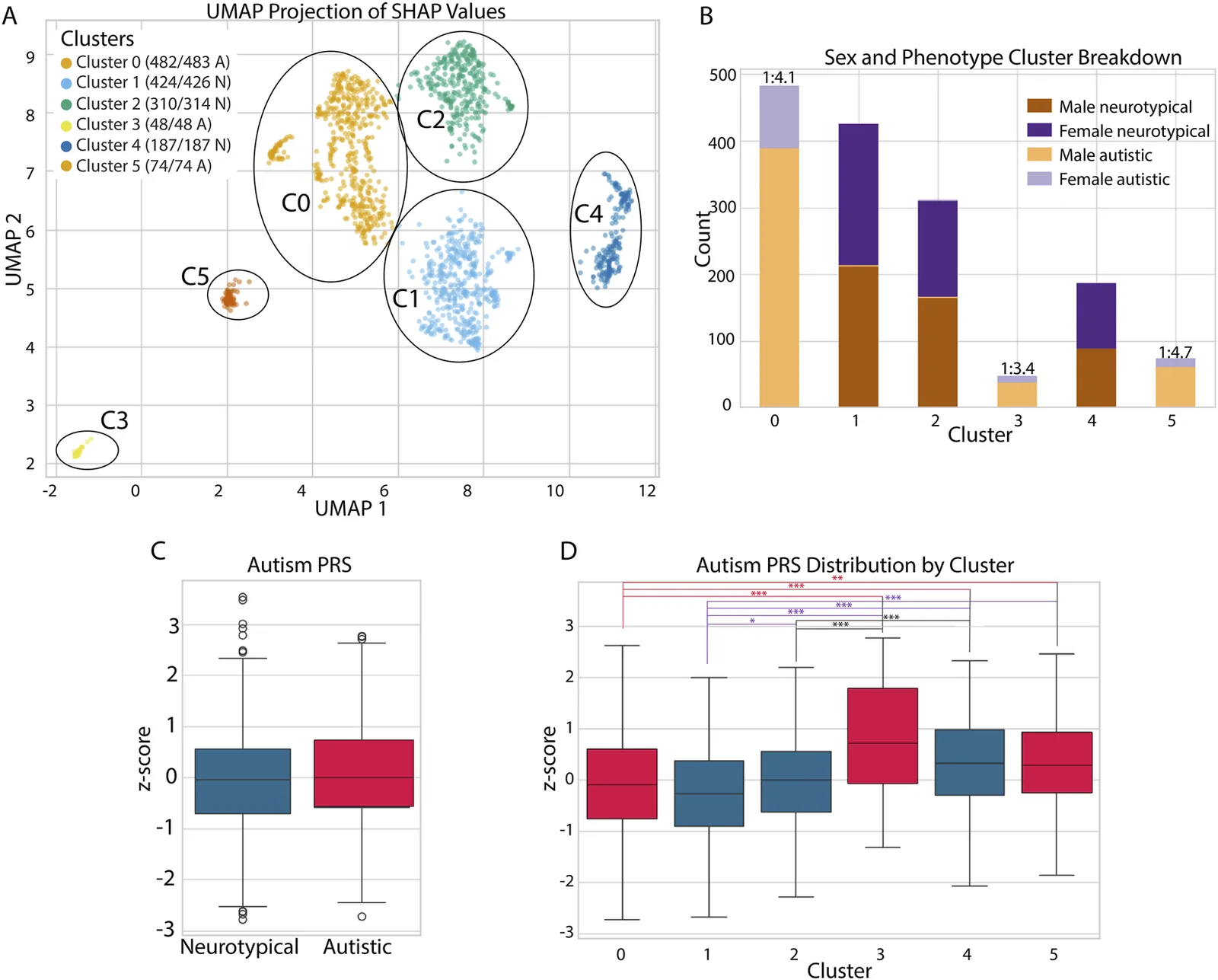
The autism polygenic risk score (PRS) can be used to cluster neurotypical and Autistic individuals. **(A)** UMAP projection of the SHAP values from autism prediction based on PRS alone. Clusters 0, 3, and 5 are comprised of Autistic individuals. **(B)** Bar graph showing the sex breakdown of the clusters with the female:male ratio listed above the Autistic clusters. There is no statistically significant difference in female:male ratio between clusters. **(C)** The distribution of autism PRS z-scores for the neurotypical and Autistic individuals. Pairwise tukey test p-value > 0.05. Z-scores calculated based on all autism PRSes. **(D)** Distribution of autism PRS z-scores per cluster. Autistic clusters are red. Comparison between clusters 1 and 2 has a p-value of 0.01, comparison between clusters 0 and 5 has a p-value of 0.006, all other p-values (***) are < 0.001.

We tested to see if it was possible to cluster Autistic and neurotypical individuals according to their genotypes, from a small number of SNPs. A random forest model was trained on all individuals using only the GWAS SNPs to predict ASD. As the purpose was clustering and no longer prediction, a separate test dataset was not required. SHAP values were used for clustering and a Uniform Manifold Approximation and Projection (UMAP) was performed to project the SHAP values into two dimensions (Figure 5A). K-means clustering (k = 6) identified three Autistic clusters (clusters 0, 3, and 5) and three neurotypical clusters (clusters 1, 2, and 4). The majority of the Autistic individuals (482/613, 79%) were present in cluster zero. There was no statistically significant difference in the female-to-male ratio between the clusters, indicating that biological sex was not a discriminating factor in the clustering model (Figure 5B). Predicted ancestry was also broadly consistent across the six clusters (85.6%–100% European; cohort-wide 89.5%; Supplementary Table S9). While the association between ancestry and cluster assignment was statistically significant, its magnitude was negligible (Cramer’s V = 0.096), likely due to the small sample size of non-Europeans.

Comparing the PRS values for all individuals, there was no statistically significant difference in the distribution between the Autistic and neurotypical groups (Figure 5C). This is likely because the neurotypical group is comprised of family members of the Autistic individuals who share common risk. However, comparisons of the PRS scores by clusters identified significant differences (pairwise Tukey p < 0.001) between clusters (Figure 5D). Notably, cluster 3 (Autistic) had the highest average PRS.

To understand the genetic features responsible for separating the clusters, we compared the presence of each SNP between each Autistic cluster and all neurotypical individuals. As was observed for overall PRS scores, no SNPs showed a statistically significant difference in presence between all Autistic and neurotypical individuals. By contrast, within cluster 0, SNPs rs112635299 and rs11787216 had a statistically significant difference in distribution compared to all neurotypical individuals (chi-squared, adjusted p-values < 0.001). SNP rs112635299 was enriched with Autistic individuals in cluster 3. SNP rs11787216 was enriched (chi-squared, adjusted p-values < 0.001) with Autistic individuals in cluster 5. These findings suggest that while no individual SNPs distinguish Autistic individuals from neurotypical individuals in this predominantly European cohort, specific genetic variations may be relevant within certain Autistic subgroups, highlighting the genetic heterogeneity within the Autistic population.

## Discussion

Our ensemble machine learning approach predicted ASD with an accuracy of 0.68 and an AUC of 0.72. The ensemble model was composed of three random forest models trained on: 1) statistically significant SNPs associated with ASD in the training dataset (Fisher SNPs), 2) ASD GWAS SNPs (Grove et al., 2019), and 3) ASD risk genes (SFARI genes listed in Supplementary Table S3), as well as biological sex. By analysing the decisions the ensemble model made, we identified genes and variants that are influential for ASD prediction. These variants included rs58741612, rs1443089352, *MTRNR2L8,* and the 16p11 locus. Collectively, these common variants contributed to prediction with greater confidence in males compared to females (accuracy = 0.69 and 0.66, respectively). Expression of sex-specific SFARI gene sets in the cohort was enriched at different developmental timepoints and in different brain regions in both males and females. These insights pave the way for more targeted and sex-informed approaches to understanding ASD genetics.

The strengths of this study lie in leveraging the comprehensive MSSNG whole-genome sequencing database (Trost et al., 2022). However, several limitations need to be considered when interpreting our findings. Firstly, the MSSNG dataset is largely European (75%) which may limit model generalisability to other ancestry groups. Because the polygenic risk scores were derived from predominantly European GWAS summary statistics, they may transfer imperfectly to individuals of other ancestries, and PRS comparisons should be interpreted in light of the cohort’s predominantly European composition. Second, while we retained the individuals who were sequenced using the Illumina platform as an independent validation dataset, a completely separately curated WGS cohort is required to test the model’s applicability to other cohorts fully. Third, our model’s exclusive focus on common genetic variation overlooks the contribution of pathogenic structural variants (SVs), which have been detected in 6% of Autistic individuals within the MSSNG database (Trost et al., 2022). Finally, SHAP values can only explain why the model predicted individuals as Autistic or neurotypical, not the ‘true’ biological causality. Given the heterogeneous nature of ASD and the complex genetic architecture involved, these predictive values should be interpreted as a proof-of-concept for the utility of ensemble methods. Phenotype data in the MSSNG database is currently sparse, meaning co-occurring conditions (such as ADHD) could not be considered during the predictive work or the clustering analysis. The primary value of this model lies in its ability to prioritise sex-dependent candidate variants for future mechanistic study, rather than as a standalone diagnostic tool.

Our ensemble model is more confident in predictions for males than for females, even after adjusting to reduce male bias. Our models identified common and X-chromosome-based variation as being important for sex-specific prediction (Figure 4A). Notably, a locus on the X chromosome has previously been associated with ASD and decreased levels of maternal serum sex-hormone-binding globulin in males, not females (Mendes et al., 2025). Our findings, which suggest that common variants contribute more to male ASD, expand on these earlier observations. It remains possible that the increased confidence in male predictions could be explained by dataset bias, specifically that females are less likely to be diagnosed (Hull and Mandy, 2017). However, the use of a balanced dataset (male:female) in our ensemble model argues against this. Therefore, we contend that our results support the hypothesis that there is a biological role for sex hormones in ASD (Mendes et al., 2025). These hypotheses should be tested further using larger, sex-balanced ASD datasets.

Social behaviour difficulties involving the prefrontal cortex manifest differently in males versus females (Laine et al., 2024; Leow et al., 2024). Autistic females tend to have more subtle and less obvious difficulties in social communication than males and are better at adapting behaviours to conform to societal norms (Ratto et al., 2018). These behavioural differences parallel sexually dimorphic patterns of brain development that are thought to contribute to the differential prevalence of neurodevelopmental conditions between males and females (Kaczkurkin et al., 2019). Consistent with sex-divergent developmental timing, the female-specific SFARI gene set in our cohort was enriched in the primary somatosensory and parietal cortices exclusively during fetal development, with no significant developmental changes across the lifespan. By contrast, expression of the male-specific SFARI genes changed significantly across development (8 post-conception weeks to 40 years) in the dorsolateral and medial prefrontal cortices (dPFC, mPFC), regions whose maturation is sex-dependent and influenced by pubertal and hormonal processes (Vijayakumar et al., 2021). This regional enrichment aligns with a recent multivariate GWAS analysis that similarly implicates cortical tissues in the shared genetic architecture of ASD and co-occurring conditions (Salenius et al., 2024). The dPFC and mPFC regions have been associated with ASD in males. The mPFC is important for social behaviour, and circuitry changes in this region have been identified in both ASD clinical studies and rodent models of majority male (Ibrahim et al., 2021; Mediane et al., 2024), or entirely male cohorts (Li et al., 2021; Mediane et al., 2024).

Notably, we identified *SHANK3* as associated with ASD in males but not females. Haploinsufficiency of *SHANK3* in Phelan-McDermid syndrome confers very high rates of ASD (over 70%; Soorya et al., 2013; De Rubeis et al., 2018). While detailed sex-stratified phenotyping of *SHANK3*-affected individuals remains limited, our male-biased association is consistent with mouse models showing that *SHANK3* mutations affect mPFC connectivity and social deficits in male but not female mice (Kim et al., 2022). Together, these sex-dependent expression windows suggest that the genetic contributors we identified act during different neurodevelopmental periods in each sex. Female-specific genes corresponded to an early sensory and parietal window, whereas male-specific genes followed a prolonged prefrontal trajectory. This timing difference may help explain why common-variant signals were more informative for males in our model, consistent with established brain-based sex differences in ASD from MRI-based human studies and animal models (Lai et al., 2017; Walsh et al., 2021). A key direction for future work will be to determine how the predictive variants identified here contribute to sex-differential neurodevelopment.

Many studies analyse the genetic risk for ASD using Autistic individuals and their family members (Klei et al., 2021; LaBianca et al., 2021; Schendel et al., 2022). Yet changes in polygenic risk scores between Autistic individuals and their neurotypical family members are, by definition, minimal. However, clustering weighted (0, 1, 2; AA, Aa, aa, respectively) GWAS SNPs overcomes this limitation. By clustering weighted PRS scores, we identified SNPs rs112635299 (associated with coronary artery disease and bronchodilation (Aherrahrou et al., 2024)) and rs11787216 (associated with educational attainment (Grove et al., 2019)) as being responsible for the greatest discriminatory difference between the Autistic and neurotypical clusters. Therefore, we hypothesise that the individuals within these clusters are more likely to have heart and education-related co-occurring traits, respectively. Notably, epidemiological studies suggest that individuals with congenital heart disease (CHD) have double the odds of developing ASD (1.99; 95% CI 1.77–2.24) than those without CHD (Gu et al., 2023). More broadly, because SNP-ASD associations vary across ancestries (Almandil et al., 2022; Pan et al., 2026), these cluster-discriminating variants reflect our predominantly European cohort and may differ in other ancestry groups.

We identified the 16p11.2 locus as important for ASD/neurotypical prediction within the MSSNG dataset, particularly for females (p-value < 0.001). The ∼600 kb deletion and reciprocal duplication at 16p11.2 are among the best-established CNV risk factors for ASD in human cohorts, together accounting for approximately 1% of cases (Weiss et al., 2008). Importantly, sex-dependent differences in diagnosis and transmission have been identified, with males with 16p11.2 deletions more likely than females to be diagnosed with ASD (Niarchou et al., 2019) and nearly 90% of *de novo* duplications and deletions arising on maternal haplotypes (Duyzend et al., 2016). Current behavioural and mechanistic evidence of sex-specific differences predominantly comes from mouse models. Specifically, male mouse models with 16p11.2 deletions are more likely to experience hyperactivity, sleep disturbances, and reduced brain sizes compared to females with the same mutation (Angelakos et al., 2017; Kretz et al., 2023). By contrast, female mouse models exhibit increased stress-induced anxiety and excitability in their cortical neurons (Giovanniello et al., 2021; Tomasello et al., 2022). However, it remains unclear what causes the sex-specific difference. Future studies on the role of the 16p11.2 locus in ASD should focus on identifying the sex-specific molecular impacts. This would provide insights that may be able to be applied to better understand the development of ASD in males versus females, potentially with significant impacts on diagnosis.

In conclusion, this study demonstrated the utility of an ensemble machine learning model trained on common genetic variants for ASD prediction. The identification of distinct male and female genetic signatures, with divergent spatiotemporal expression patterns and developmental trajectories, provides compelling evidence for sex-specific etiological pathways on ASD risk. Future work should explore the mechanisms underlying the sex-specific effects of the 16p11 locus.

## Data Availability

Table S5 lists the datasets and software that were used in our analyses. All scripts are available on GitHub (https://github.com/Catriona-Miller/autism_ml).
