# Supplementary material for: Sex-dependent prediction of autism
Source: Front Genet. 2026 Aug 13;17:1799530. doi: 10.3389/fgene.2026.1799530 (PMC13516016; doi:10.3389/fgene.2026.1799530)
Supplement: Supplementary file 1 [file Supplementaryfile1.docx]

**Supplementary Figure S1:** Pipeline used to select the 790 Fisher SNPs for the first machine learning model. Code can be found at https://github.com/Catriona-Miller/autism_ml/

**Supplementary Figure S2:** The ensemble model achieves a recall of 0.77 and a precision of 0.61. Recall is the proportion of Autistic individuals (TP+FN; 136) who are correctly predicted to be Autistic (TP; 105). Precision is the proportion of individuals predicted to be Autistic (TP + FP; 171) who are Autistic (TP; 105).

**Supplementary Figure S3**: Common variants before the model was balanced were largely sex-specific. Phenogram showing the top 10% of common variants for prediction in the original, unbalanced model. Blue dots were only in the top 10% of variants for females, green dots were only in the top 10% of variants for males, and red dots were in the top 10% of variants for males and females.

**Supplementary Figure S4**: SFARI genes that have a significant difference in mutation count between Autistic and neurotypical individuals. (A) Venn Diagram showing the number of SFARI genes that are significantly more/less mutated in Autistic individuals than neurotypical for males and females. (B-D) Pie chart showing the chromosomes containing the genes that had significantly different mutation rates between neurotypical and Autistic individuals in females (B), both males and females (C), and males (D). (E)&(F) LOEUF scores of SFARI genes that had significantly different numbers of mutations in males (E) and females (F) compared to those that were insignificant for males and females. p values < 0.001. (G) The areas of the brain that were significantly enriched in the gene expression of the female genes (n=159) or male genes (n=579) at different developmental timepoints, from the Allen Brain Atlas. DFC and MFC have significant developmental changes in gene expression for the male gene list. M1C: primary motor cortex, CBC: cerebellar cortex, OFC: orbital frontal cortex, IPC: posteroventral (inferior) parietal cortex, VFC: ventrolateral prefrontal cortex, STC: posterior (causal) superior temporal cortex, S1C: primary somatosensory cortex, PCx: parietal neocortex, DFC: dorsolateral prefrontal cortex, MFC: anterior (rostral) cingulate (medial prefrontal) cortex.
